# Military Magic or Nature’s Fool

**DOI:** 10.3201/eid1804.AC1804

**Published:** 2012-04

**Authors:** Polyxeni Potter

**Affiliations:** Centers for Disease Control and Prevention, Atlanta, Georgia, USA

**Keywords:** art science connection, emerging infectious diseases, art and medicine, Charles Burchfield, Camouflage, vector-borne infections, American art, military magic or nature’s fool, about the cover

**Figure:**
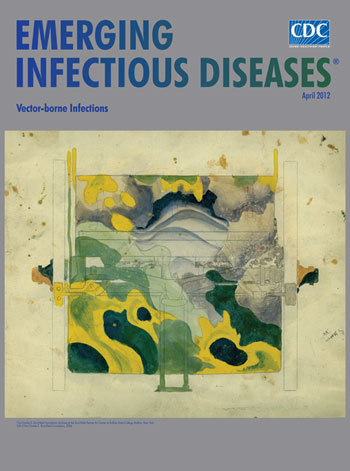
**Charles E. Burchfield (1893–1967) *Camouflage design* (1918) Watercolor and graphite on paper mounted on black paper (27.3 cm x 32.1 cm)** The Charles E. Burchfield Foundation Archives at the Burchfield Penney Art Center at Buffalo State College, Buffalo, New York. Gift of the Charles E. Burchfield Foundation, 2006

“If this world lasts for a million years or two million years, or more, never can you exhaust the subject matter of humanity and nature,” said Charles Burchfield regarding his source of inspiration. “I don’t know how much time I’ve got left, but I’d like to have at least another lifetime… to say what I want to say about nature.” This fascination began during the artist’s childhood in Salem, Ohio, where he “formed the habit of wandering off to the woods and fields… in search of wild flowers in the spring or colored leaves in the fall.” These, along with blossoming trees and all manner of plants, made up his earliest drawings, also described in copious journals, more than 10,000 pages. “I hereby dedicate my life and soul to the study and love of nature, with the purpose to bring it before the mass of uninterested public.”

Burchfield’s art education began at the Cleveland School of Art, where he became familiar with the teachings of artist and philosopher Arthur Wesley Dow who, ahead of his time, believed that nature should be depicted not in realistic terms but in a harmonious presentation of compositional elements (line, color, light and dark). During these years, Burchfield absorbed such diverse influences as Chinese scroll paintings; the works of Hiroshige, Hokusai, and Aubrey Beardsley; and Russian ballet designs by Léon Bakst. But “The greatest inspiration to me was Henry G. Keller… not only a good painter but also a good teacher…. He made you feel as though art was the most important thing in the world, and you couldn't do better than to be an artist if you had the aptitude for it.” Keller said that his student’s “inability to see form” and his “virtually complete concentration on two-dimensional pattern amounted almost to genius.”

A scholarship in 1916 to the national Academy of Design in New York City did not appeal, as Burchfield dropped out of the program after 1 day. But during his brief stay in the city, he met art critic and instructor Mary Mowbray-Clarke, who showed his works at her gallery, the Sunwise Turn Book shop, launching his career as a leading artist. Later, he was to describe 1917 as his “golden year.” This period and up to 1918 was also his most prolific. This was too a time of war, so he joined the army. “I was made a sergeant and I had a half a dozen men under me, and we were doing camouflage. And I was happy making the designs for camouflage.”

After this brief career in the military, he returned to Salem, where he continued to paint around his work as accountant in an auto parts company. When a better opportunity came up, he moved to Buffalo, New York, to work as designer for H. M. Birge and Sons, “the finest wallpaper firm in the country.” “I had to make a living somehow…. I knew it would be years before I could hope to make any money out of painting.” It was 1929 and his work was shown in the prestigious Montross Gallery when he made the decision to devote all his time to painting.

Burchfield felt most comfortable with the medium of watercolor. “I use a dry paper and what is called a dry brush, which isn’t dry, of course, in that it has the minimum amount of water in it, and I stand them up… just as a man painting an oil painting, except that you are using different materials.” Of his technique, he said, “I like to advance and retreat, just like a man writing a book. I doubt that very few of them ever sit down and leave a paragraph as it first comes into their head. They work over it, delete things and add things…. A picture has a life and direction of its own, and the artist has to find out which way the picture wants to go and follow …. It sounds like doubletalk, but it’s true.”

*Camouflage design*, on this month’s cover, one of two surviving studies from Burchfield’s military days, is a mix of abstract and realistic elements and like all his work reaches for more than meets the eye. At home with modern camouflage, he built on art invented by those like him, who studied nature. American naturalist and artist Abbott Handerson Thayer (1849–1921), sometimes referred to as the father of camouflage because of his pioneering research on protective coloration in nature, laid out basic principles: the color of a camouflaged object must match its background. Strong patterns work better than delicate ones because they interfere with how we read the outline of a form. A dark strip at the top and a white splash underneath disrupt our expectations of how an object will appear (countershading).

Crude forms have been used in warfare since antiquity. Shakespeare knew camouflage. In Macbeth, at the wood of Birnam, Malcolm said, “Let every soldier hew him down a bough / And bear’t before him: thereby shall we shadow / The numbers of our host and make discovery /Err in report of us.” And then, “As I did stand my watch upon the hill, / I look’d toward Birnam, and anon, methought, / The wood began to move.”

Thayer’s ideas were quickly put to military and artistic use. And when the United States entered World War I, several artists, Burchfield among them, became involved, producing various patterns, initially representational then boldly abstract. When cubism swept the art scene, obliterating the distinction between an object and its background, it influenced military camouflage designs. In The Autobiography of Alice B. Toklas, Gertrude Stein described an evening in 1915, when she and Toklas were strolling with Picasso and his mistress Eva Gouel. “All of a sudden down the street came some big cannon, the first any of us had seen painted, that is, camouflaged. Pablo stopped, he was spell-bound. ‘*C’est nous qui avons fait ça*,’ he said.”

Near the end of his life, Burchfield went back to the paintings of his golden year, his time most inspired by nature. He pasted them into new works, seamlessly expanding and absorbing them into larger creations incorporating the most profound discoveries of his later artistic career. These masterpieces, the culmination of his love for nature, seem to complete his desire to become one with it.

The merger between art and nature sensed by camouflage artists has broad applicability in disease emergence, where the borders, geographic and biologic, blend as smoothly and seamlessly as in art. Burchfield’s *Camouflage design* with its graceful curves, flat patterns, and strong colors over linear designs is a perfect portrait of natural elements, overlapping and filled with movement, light, or heat, the underpinnings as well as the surface of nature, and most of all, its mystery and intrigue.

A look at the ecologic dynamics, meteorologic variables, and transmission cycles, along with human travel and vector control of malaria alone as it reemerges around the world, attests to Burchfield’s acute understanding of complexity underneath the surface. In Ecuador, the railway inadvertently provided transportation of the vector and parasite to higher elevations. In other parts of the world, dengue virus activity, a major cause of illness in travelers, is increasing and, until an effective vaccine is licensed, will likely remain a threat to military troops operating where the disease is endemic. Recent outbreaks in Hawaii, Texas, and Florida resulted in recommendations on public health response, including such vector control activities as spraying for adult mosquitoes and eliminating standing water around homes. In Veneto Italy, surveillance to identify cases of West Nile fever, imported dengue and chikungunya infections in travelers also highlighted, among other measures, the need for vector control. But, while artists and the military have managed to fool human enemies by camouflage, we have yet to adequately fool mosquito and other vectors of disease.
